# Fetal Adrenal Suppression Due to Maternal Corticosteroid Use: Case Report

**DOI:** 10.4274/jcrpe.v3i3.31

**Published:** 2011-09-09

**Authors:** Selim Kurtoğlu, Dilek Sarıcı, Mustafa Ali Akın, Ghaniya Daar, Levent Korkmaz, Şeyma Memur

**Affiliations:** 1 Erciyes University Faculty of Medicine, Department of Pediatrics Division of Neonatology, Kayseri, Turkey; 2 Nevşehir Government Hospital, Deparment of Pediatrics, Nevşehir, Turkey; +90 535 255 82 55 drleventkorkmaz@yahoo.com

**Keywords:** Pregnancy, exsogenous corticosteroids, fetal adrenal suppression

## Abstract

During pregnancy, steroids are usually used in maternal diseases  such as adrenal failure or other autoimmune diseases, e.g. idiopathic thrombocytopenic purpura (ITP), Crohn’s disease, systemic lupus  erythematosus, dermatomyositis, scleroderma, Addison’s disease and hyperemesis gravidarum, HELLP syndrome. Endogenous or exogenous maternal steroids are metabolized by the placental enzyme 11  beta-hydroxy steroid dehydrogenase type 2. Prednisolone and  methylprednisolone are highly sensitive to this enzyme, while  dexamethasone and betamethasone are less well metabolized. Steroids which can cross the placental barrier are administered in cases like fetal lupus, congenital adrenal hyperplasia and for enhancement of fetal lung maturation, whereas steroids used in maternal diseases are usually the ones with low affinity to the placenta; however, in case of long-term use or in high doses, placental enzyme saturation occurs and thus, resulting in fetal adrenal suppression. Antenatal steroids can lead to low birth weight, as observed in our patient. Here, we report a case with fetal adrenal suppression due to maternal methylprednisolone use presenting with early hypoglycaemia and late hyponatremia in neonatal period and requiring three-month replacement therapy.

**Conflict of interest:**None declared.

## INTRODUCTION

Corticosteroids administered during pregnancy or maternal Cushing’s syndrome can cause suppression of fetal adrenal glands (1,2,3,4,5,6,7). Maternal use of corticosteroids is  needed in case of fetal congenital adrenal hyperplasia, as well as in maternal diseases such as idiopathic thrombocytopenic purpura (ITP), Crohn’s disease, systemic lupus erythematosus, Addison’s disease and rheumatological problems (3,8,9).  Short-term corticosteroid treatment is given in case of preterm labor to enhance fetal lung maturation (8). When using  corticosteroids during pregnancy, the choice of preparation type and dose is of utmost importance - steroids crossing the placenta freely should be given if the target is fetus,  while those  passing across the placenta should be used in smaller amount if maternal disorders are being treated (1).

 In this article, adrenal suppression pattern in a newborn exposed to long-term maternal methylprednisolone therapy were presented with special emphasis on short term follow up of such infants.  

## CASE REPORTS

A 20-minute-old newborn, whose mother used 64 mg methylprednisolone per day during her pregnancy due to ITP, was hospitalized for follow-up. Pregnancy duration was 39 weeks. The neonate was 2680 grams (3-10th percentile) at birth with head circumference of 36 cm (75-90th percentile) and height of 50 cm (25-30th percentile). Whole blood examination showed hemoglobin level of 19.1 g/dL, leukocyte count of 10 530/mm3, and platelet count of 10 000/mm3. Biochemistry  profile revealed the following: blood glucose 29 mg/dL,  sodium level 138 mEq/L, potassium 4.5 mEq/L, ALT 18 U/L, AST 62 U/L, calcium 9.5 mg/dL, phosphorus 4.8 mg/L, alkaline phosphatase 100 U/L, parathormone 13.91 pg/mL. On adrenal ultrasonographic examination, the adrenal glands were small measuring 10x2 mm in size for the right and 12x2 mm for the left one. Since the patient was thrombocytopenic, 0.8 g/kg IVIG infusion was given and repeated platelet count was 30 000 mm3. On the third day of follow-up, sodium level became 123 mEq/L, potassium 3.9 mEq/L, and urinary sodium level was 26 mEq/L. On the fourth day, cortisol level was 16.22 μg/dL, ACTH was 44.4 pg/mL, 17-OH progesterone was 2.58 ng/mL. On the 10th day, rechecking the adrenal functions,  cortisol level was found to be 0.194 μg/dL and ACTH 20.9 pg/mL. After administration of 1 μg of ACTH i.v. (low-dose ACTH test), the cortisol level increased to 9.69 μg/dL at 30 minute. Then, the patient was given 3 mg/m2/day p.o.  methylprednisolone as physiological replacement. The result of low-dose ACTH test on the 40th day postpartum was as  follows: basal cortisol level of 4.29 μg/dL and 30th minute  cortisol level of 11.29 μg/dL. Therefore, methylprednisolone therapy was continued  and stopped by slowly tapering at the end of the 3rd month (Table 1). Low-dose ACTH test was repeated in the 4th posnatal month  and the results were as follows: basal cortisol 4.75 μg/dL, ACTH 19.5 pg/dL. After 30 minutes, cortisol level was 19.9 μg/dL. Hormone tests and their results are summarized in Table 1. These results showed that the patient was relived from adrenal suppression.  

**Table 1:**
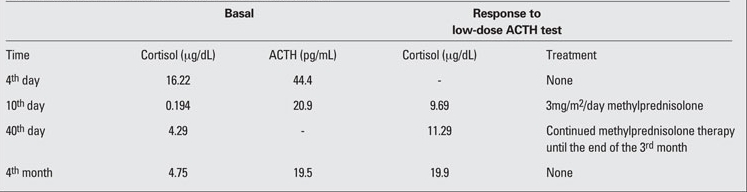
Table 1. Hormone levels and treatment of the patient during follow-up

## DISCUSSION

Corticosteroids are given during pregnancy if needed in maternal diseases or other pregnancy-related problems as well as to treat certain fetal diseases; in the latter cases,  corticosteroids capable of crossing the placenta are  administered to the mother (5,6,7,8,9). 

As side effects to the mother, steroids used during  pregnancy can cause weight gain, dyslipidemia,  hypertension, cushingoid appearance, acne, hypertrichosis, psychological problems (8). Corticosteroids are metabolized in the placenta by the help of the enzyme 11-b-hydroxylase steroid dehydrogenase-2 (10). Cortisol, a physiologic steroid, is metabolized to cortisone. Similarly, synthetic  glucocorticosteroids are metabolized to inactive  metabolites in the placenta. Prednisolone-related drugs are mostly degraded to inactive forms by the placental enzymes and, approximately 10% of the total amount will ultimately reach the fetus and among this whole, about 33% of betamethasone and 50% of dexamethasone will enter the fetal circulation (11,12).  Besides this, when taken in high doses and for long period of time, prednisolone and  methylprednisolone themselves can saturate the placental enzymes and, as a result, large amount of corticosteroids can cross the placental barrier causing significant  suppression of the fetal glands, as observed in our case (5). Fetal adrenal suppression develops approximately within 14 days after maternal steroid use, therefore, the neonate may be born with ACTH suppression (5). Adrenal gland  insufficiency becomes prominent on postnatal day  3 - the neonate develops hyponatremia, hypoglycemia and hypotension. Since there is central adrenal insufficiency due to long-term steroid effect, potassium level is within normal limits, or even low. It is well known that  long-term steroid use can cause low birth weight, as in  our case (12). 

 In our patient, high-dose methylprednisolone saturated the placental enzymes, the steroids crossed the placenta more significantly and in higher amounts, thus, causing fetal adrenal suppression.  On the fourth day, cortisol level was within normal ranges, but we consider that there might be an  interference between crossed maternal steroids, their  metabolites and fetal cortisol. Since on the 10th day ACTH and cortisol levels were found to be suppressed, this shows the importance of measuring cortisol and ACTH levels during the second week. 

 Thus, steroids crossing the placenta in small amount should be preferred during pregnancy in case of maternal disorders necessitating steroid use. The newborns should be followed postnatally. On postnatal day 4, basal cortisol and ACTH levels should be measured, and if needed, adrenal reserves should be checked by conducting low-dose ACTH test. Although there are many references for threshold of cortisol response to low-dose ACTH test, for term newborns, levels of 20 μg/dL and above should be accepted as normal response (13). 

 For patients with adrenal insufficiency, physiological replacement should be started. Moreover, in case of stressful conditions, the dose of steroid should be increased 2-3 times, because it has been shown that antenatal steroids can change the response to neonatal stress (14). Low-dose ACTH test should be repeated at specified intervals and, as soon as  adrenal response returns to normal, replacement therapy should be slowly tapered and stopped. 
